# Effect of *Phyllanthus amarus* on serum biochemical changes in azaserine induced pancreatic cancer in Wistar rats

**DOI:** 10.14202/vetworld.2015.937-940

**Published:** 2015-08-07

**Authors:** Ankit S. Prajapati, Sunant K. Raval, Suprita Sinha, Tapan N. Varia, Parimal H. Mashiyava

**Affiliations:** 1 Department of Veterinary Medicine, College of Veterinary Science and Animal Husbandry, Anand Agricultural University, Anand, Gujarat, India; 2 Department of Veterinary Pharmacology and Toxicology, College of Veterinary Science and Animal Husbandry, Anand Agricultural University, Anand, Gujarat, India

**Keywords:** azaserine, pancreatic cancer, *Phyllanthus amarus*, serum biochemistry, Wistar rat

## Abstract

**Aim:**

The present study was performed to investigate the effect of *Phyllanthus amarus* extracts on serum biochemical changes in azaserine induced pancreatic cancer in Wistar rats.

**Materials and Methods:**

Pancreatic cancer was developed in Wistar rats by intraperitoneal administration of azaserine (cancer inducer) for 21 days at the concentration of 5 mg/kg body weight. Aqueous and alcoholic extracts were given to rats of different groups as per protocol.

**Results:**

The results data revealed that oral administration of *P. amarus* extracts had a significant change in pancreatic amylase, lipase, aspartate aminotransferase, alanine aminotransferase, and alkaline phosphatase activity.

**Conclusion:**

We concluded that extract of *P. amarus* possessed chemoprotective activity against azaserine induced pancreatic cancer in Wistar rats.

## Introduction

Cancer is a devastating disease with a severe impact on the physical and psychological well-being of the patient. Pancreatic cancer is one of them. Cases of pancreatic cancer have been reported in various species of animals, including dogs and cats. Forty-seven percentage of dogs and 32% of cats over 10 years of age died of cancer [1].

No specific treatment options are available for such diseases until date. It is necessary to find out alternative therapeutic options to treat or cure such conditions. This leads to a search for alternative therapies, including the holistic approach of alternative medicine, especially preparations from herbal products, which have formed the basis for traditional medicine for thousands of years. The plant has been used in the treatment of cancer [2].

*Phyllanthus amarus* is a well-known plant in Ayurveda and Siddha [3]. Ayurveda describes it to be a potent drug against a variety of ailments and is used for problems of the stomach, liver, kidney, spleen, and pancreas [4]. It possesses a lot of medicinal properties particularly anti-cancerous or chemoprotective activity [5]. *P. amarus* has been used to treat flu, cancer, dropsy, diabetes, and jaundice [6]. Extract of *P. amarus* contains so many active components in it which exerts anti-oxidant activity and prevent tumor growth [7,8]. Biosynthesis of these active compounds depends on the environment in which they grow [9]. *P. amarus* possesses anti-oxidant, hepatoprotective, anti-fertility, anti-diarrheal, anti-spasmodial, anti-tumor, chemoprotective, antiviral, and antidiuretic properties [10-13]. *P. amarus* preparations or extract is known or reported to treat liver disorders [14].

Azaserine, used in this study is a well-known potent pancreatic-carcinogenic agent that has been reported to generate free radicals which exert its carcinogenic effects, and has been widely used in pancreatic carcinogenesis in experimental rats [15].

Animal models are used for the *in vivo* study of different chemicals and drugs [16,17]. Azaserine induced cancer rat model is ideal for the pancreatic cancer study [18]. A wide range of medicinal plants are available, but their use is still unknown, so *P. amarus* extract is being evaluated for its use in the prevention of pancreatic cancer in the rat.

## Materials and Methods

### Ethical Approval

All the protocols as per the Committee for the Purpose of Control and Supervision of Experiments on Animals guidelines on the care and use of Laboratory animals were followed and approved by the Institutional Animal Ethics Committee of Veterinary College, Anand, Gujarat, India.

### Study area

The study was conducted at the Department of Veterinary Medicine with the help of Department of Veterinary Pharmacology and Toxicology, Veterinary Pathology and Department of Livestock Production and Management, College of Veterinary Science and Animal Husbandry, Anand Agricultural University, Anand, Gujarat, India.

### Animals

Eighty healthy adult Wistar rats (either sex) of 8-12 weeks of age were procured from Zydus Research Centre (ZRC), Moraiya, Ahmedabad, Gujarat.

### Plant

*P. amarus* plants were procured from Medicinal and Aromatic Plant Unit, Anand Agricultural University, Anand. Identification and authentication were also done by people of Medicinal and Aromatic Plant Unit, Anand Agricultural University, Anand, Gujarat, India.

### Preparation of aqueous and alcoholic extract *P. amarus*

Plant of *P. amarus* was taken and dried under shade, then powdered by the mechanical grinder, sieved, and stored in airtight containers. Exactly 100 g of coarsely powdered material of *P. amarus* was successfully extracted in Soxhlet Extractor with water and also with alcohol. Extracts so obtained were decanted in a beaker and then concentrated to 1/6^th^ of total volume in a water bath. The aqueous and alcoholic extracts were preserved in the refrigerator.

The research was carried out on the toxicity of aqueous and alcoholic extracts of *P. amarus*. The study stated that it is safe to give extract up to 400 mg/kg body weight has no toxic effect on the animal. The study provides pivotal evidence for ascertaining the safety of the standardized (LD_50_ > 5000 mg/kg) that could be used as tonic or food supplement in medicine. So, we kept our dose slightly lower and in two upward level i.e. 200 mg/kg and 400 mg/kg body weight for our study [19].

### Experiment protocol

Rats were selected randomly and divided into eight groups (Groups I, II, III, IV, V, VI, VII and VIII). All Groups had ten animals each. All the rats were numbered group wise and individually. Group I served as healthy control consisted of healthy animals. Pancreatic cancer was induced in Group II animals using azaserine at the dose rate of 5 mg/kg body weight as cancer-inducing agent. Group II animals were kept untreated. After 1 h fasting, animals of Groups III, IV, V, and VI were administered 5 mg/kg intraperitoneally azaserine by dissolving it in distilled water, once in a week for 3 weeks and then after 1 h all those rats were administered test compounds. Aqueous and alcoholic extracts of *P. amarus* were dispersed in water and administered to animals of Groups III, IV, V, and VI. Group III animals were administered aqueous extract of *P. amarus* at dose rate of 200 mg/kg body weight and Group IV animals were administered aqueous extract of *P. amarus* at dose rate of 400 mg/kg body weight. Group V animals were administered alcoholic extract of *P. amarus* at a dose of 200 mg/kg body weight, and Group VI animals were administered extract of *P. amarus* at a dose rate of 400 mg/kg body weight. Animals of Groups VII and VIII were administered aqueous and alcoholic extract at a dose rate of 400 mg/kg, respectively as extract control. The extracts were administered to rats directly in the esophagus by using rat oral feeding needle with 2 ml BD syringe for 21 days.

### Biochemical analysis

The blood samples were collected, and serum was separated on the 22^nd^ day. Serum biochemical parameters (alanine aminotransferase [ALT], aspartate aminotransferase [AST], alkaline phosphatase [AKP], albumin, amylase, and lipase) were estimated using standard assay kits (Coral Clinical System, Goa, India) with the help of clinical serum biochemistry analyzer (Photometer 5010 V5+, Dynalab Enterprize).

### Statistical analysis

One-way analysis of variance was used to compare the effects of *P. amarus* extracts with normal control group, azaserine control group and groups given plant extract on biochemical parameters by using software SPSS (Version 20; Armonk, NY: IBM Corp, USA).

## Results

The effect of administration of *P. ­amarus* extract on serum activity of AST, ALT, AKP, Albumin, pancreatic amylase, and lipase are shown in Table-1. Significant changes were observed in the activity of pancreatic amylase, lipase, and AKP. The level of pancreatic amylase in Groups I and II was 2251.30±136.21 U/L and 2684.40±73.48 U/L, respectively. In Group, II level was significantly (p≤0.05) increased than the normal control group. It indicates that the azaserine has created an effect on the pancreatic cell, and it leads to increasing amylase level. In Groups III and IV amylase value was 2317.00±170.48 U/L and 2304.80±166.62 U/L, respectively. Groups V and VI were given alcoholic extract 200 mg/kg body weight and 400 mg/kg body weight respectively. The amylase level was 2251.00±135.37 in Group V and it was 1963.70±166.09 in Group VI. In Group VII value was 1959.50±119.03 U/L and in Group VIII value was 2168.90±99.06 U/L.

**Table-1:** Effect of *Phyllanthus amarus* extract on serum biochemical changes in different groups of wistar rats (mean±SE).

Groups	Mean±SE (n=80)					

	ALT (U/L)	AST (U/L)	AKP (U/L)	Albumin (g/dl)	Pancreatic amylase (U/L)	Lipase (U/L)
I	191.60±9.39	202±8.58^a^	20.10±1.22^ab^	4.2±0.20	2251.30±136.2^a^	46.10±1.72^a^
II	200.90±12.41	240±8.16^b^	39.50±2.29^d^	3.3±0.26	2684.40±73.48^b^	76.10±1.32^b^
III	187.00±18.96	215±7.93^a^	19.80±1.75^a^	4.1±0.23	2317.00±170.48^ab^	55.50±3.18^a^
IV	193.00±14.64	213±8.94^a^	22.50±0.95^abc^	3.7±0.21	2304.80±166.62^ab^	53.30±5.77^a^
V	212.50±27.88	203±7.68^a^	24.90±1.21^bc^	3.8±0.13	2251.00±135.37^a^	53.10±4.44^a^
VI	200.00±9.98	205±7.20^a^	27.30±2.82^c^	4.0±0.21	1963.70±166.09^a^	53.30±6.78^a^
VII	211.10±15.02	210±8.16^a^	19.60±0.76^a^	3.8±0.20	1959.50±119.03^a^	53.30±6.78^a^
VIII	190.20±11.32	210±8.16^a^	19.40±0.89^a^	3.9±0.31	2168.90±99.06^a^	50.90±2.86^a^

SE=Standard error, ALT=Alanine aminotransferase, AST=Aspartate aminotransferase, AKP=Alkaline phosphatase. Mean value with single superscript in a column vary significantly at p<0.05

The level of lipase in Group I was 46.10±1.72 U/L and in Group II 76.10±1.32 U/L. In Group, II level was significantly (p<0.05) increased than the normal control group. It indicates that azaserine has created an effect on the pancreatic cell, and it leads to increasing in lipase level. In Groups III and IV the lipase level was 55.50±3.18 U/L and 53.30±5.77 U/L respectively. Groups V and VI were given 200 and 400 mg/kg body weight alcoholic extract respectively. The lipase level was 53.10±4.44 U/L in Group V and 53.30±6.78 U/L in Group VI. AKP value was also significantly increased in Group II and in extract treated group it was near to a normal value.

## Discussion

Despite recent advances in our understanding of the biological processes leading to the development of cancer, there is still a need for new and effective agents to keep this disease under control [20]. In the recent times, focus on plant research has increased all over the world, and a large body of evidence has collected to show the immense potential of medicinal plants used in various traditional systems [21].

In the present study, aqueous and alcoholic extract of *P. amarus* was evaluated for effect on serum biochemical changes to know its chemoprotective activity. Data suggest that *P. amarus* can be used for prevention of pancreatic cancer. Amylase and lipase are important enzymes which help in digestion of starch and fat. Pancreatic amylase completes digestion of carbohydrate, producing glucose, a small molecule that is absorbed into the blood and carried throughout the body for energy supply. Pancreatic lipase acts on these fat globules, converting them into fatty acids and glycerol, which are small, energy-dense molecules used by all cells. Fatty acids and glycerol travel in blood and lymph vessels to reach all parts of the body.

In cancer, cell proliferation occurs, and it leads to more enzyme secretion. *P. amarus* administration to azaserine treated rats with alcoholic extract (Groups V and VI) restore amylase level to near control levels (Group I). It indicates that *P. amarus* exhibit anti-proliferative activity against pancreatic cancer and thus suggesting that it may be able to regulate cell proliferation at G_1_ phase. Lipase is also associated with pancreas activity. Increase level of lipase in Group II indicates the damage of pancreatic cells. In Groups III, IV, V and VI such damage was prevented because these groups were treated with plant extract. It indicates the protective action of plant extract against pancreatic damage. High AKP value can be observed in liver related conditions. *P. amarus* has also activity against liver associated disorders. In groups other than cancer control AKP value was near normal.

*P. amarus* possesses an antioxidant activity that may be responsible for its free radical scavenging ability and thus preventing tumor promotion [22,23]. Recent data have expanded the concept that inflammation is a critical component of tumor progression. Macrophages induce the generation of reactive oxygen species (ROS) within tumor cells through secretion of various stimuli, such as tumor necrosis factor alpha. Production of ROS by neutrophils and macrophages as a mechanism to kill tumor cells is well established. In these cells, a rapid burst of superoxide formation primarily mediated by nicotinamide adenine dinucleotide phosphate oxidase leads to the subsequent production of hydrogen peroxide. Furthermore, during inflammation processes, activated macrophages also generate nitric oxide which reacts with superoxide to produce peroxynitrite radicals that are similar in their activity to hydroxyl radicals and contribute to tumor cell apoptosis. Furthermore, it is a rich source of phytochemicals such as flavonoids, phenols, tannins, and polyphenols (two of which e.g. quercetin and ellagic) enhance the antioxidant defense in azaserine induced pancreatic cancer [24]. Oyewo *et al*. [25] reported that the aqueous leaf extract of *P. amarus* can be used as blood tonic for the prevention and/or cure of infective and degenerative diseases.

## Conclusion

The study revealed that *P. amarus* extract work as an anti-oxidant agent and thereby prevent cell proliferation. Extract of *P. amarus* possesses chemoprotective activity. Our study revealed that *P. amarus* extract can be given as a supplement to as a chemoprotective agent. Synthetic drugs have so many side effect and on the other hand, medicinal plants can be used with no or minimum side effects.

## Authors’ Contributions

This study is the major component of the work toward the M. V. Sc. thesis of the first author ASP. SKR: Provided guidance during the entire experiment and corrected manuscript. SS, TNV, and PHM: Helped in blood collection from rats and biochemical analysis. All authors have read and approved the final version of the manuscript.
